# The combined effect of the T2DM susceptibility genes is an important risk factor for T2DM in non-obese Japanese: a population based case-control study

**DOI:** 10.1186/1471-2350-13-11

**Published:** 2012-02-24

**Authors:** Kimiko Yamakawa-Kobayashi, Maki Natsume, Shingo Aoki, Sachi Nakano, Tomoko Inamori, Nobuhiko Kasezawa, Toshinao Goda

**Affiliations:** 1 Laboratory of Human Genetics, School of Food and Nutritional Sciences, Graduate School of Nutritional and Environmental Sciences, Global COE Program, University of Shizuoka, Shizuoka 422-8526, Japan; 2 Laboratory of Nutritional Physiology, School of Food and Nutritional Sciences, Graduate School of Nutritional and Environmental Sciences, Global COE Program, University of Shizuoka, Shizuoka 422-8526, Japan; 3 Department of Data Managements for Health Evaluation & Promotion, Shizuoka Medical Center, Shizuoka 422-8033, Japan

## Abstract

**Background:**

Type 2 diabetes mellitus (T2DM) is a complex endocrine and metabolic disorder. Recently, several genome-wide association studies (GWAS) have identified many novel susceptibility loci for T2DM, and indicated that there are common genetic causes contributing to the susceptibility to T2DM in multiple populations worldwide. In addition, clinical and epidemiological studies have indicated that obesity is a major risk factor for T2DM. However, the prevalence of obesity varies among the various ethnic groups. We aimed to determine the combined effects of these susceptibility loci and obesity/overweight for development of T2DM in the Japanese.

**Methods:**

Single nucleotide polymorphisms (SNPs) in or near 17 susceptibility loci for T2DM, identified through GWAS in Caucasian and Asian populations, were genotyped in 333 cases with T2DM and 417 control subjects.

**Results:**

We confirmed that the cumulative number of risk alleles based on 17 susceptibility loci for T2DM was an important risk factor in the development of T2DM in Japanese population (*P *< 0.0001), although the effect of each risk allele was relatively small. In addition, the significant association between an increased number of risk alleles and an increased risk of T2DM was observed in the non-obese group (*P *< 0.0001 for trend), but not in the obese/overweight group (*P *= 0.88 for trend).

**Conclusions:**

Our findings indicate that there is an etiological heterogeneity of T2DM between obese/overweight and non-obese subjects.

## Background

Type 2 diabetes mellitus (T2DM) is a complex endocrine and metabolic disorder. The interaction between multiple genetic and environmental factors is considered to contribute to the pathogenesis of the disease [1-3]. Most patients with T2DM suffer serious complications due to chronic hyperglycemia, including nephropathy, neuropathy, retinopathy and accelerated development of cardiovascular disease.

The prevalence of type 2 diabetes is continuing to increase in many countries, including Japan [4,5]. Clinical and epidemiological studies have indicated that obesity is a major risk factor for T2DM, because obesity is associated with an increased risk of developing insulin resistance and impaired glucose tolerance [6,7]. When β-cells are no longer able to secrete sufficient amounts of insulin to overcome insulin resistance, impaired glucose tolerance progresses to T2DM [7].

The prevalence of obesity is also increasing worldwide, although there are large ethnic differences in the degree of obesity reached. In Japan, the prevalence of severe obesity (BMI ≥ 30 kg/m^2^) is lower than that in Western countries, where the prevalence of overweight (25 ≤ BMI < 30) adults has been steadily increasing [5,8]. It is thought that genetically, insulin secretion compensation for insulin resistance is weaker in the Japanese than in Caucasians, and thus, being mildly overweight also conveys a risk factor for diabetes in Japan [8]. In fact, the prevalence of T2DM in the Japanese population is as high as in Western countries, although the prevalence of obesity is much lower than that seen in Caucasians [4,5,8,9].

Recently, several genome-wide association studies (GWAS) have identified many novel susceptibility genes for T2D. To date, approximately 40 susceptibility loci for type 2 diabetes have been identified [10-17]. Most of these susceptibility loci were detected in Caucasians, and they have been widely confirmed to be susceptible loci for T2DM in Asian populations [17-23]. Furthermore, additional GWAS for Japanese and Chinese populations were performed and new susceptibility loci were detected [24-28], some of which have also been confirmed in Caucasians as well as Asian populations [24,25,29]. Thus, GWAS indicate that there are common genetic causes contributing to the susceptibility to T2DM in multiple populations worldwide, although clinical risk factors, such as BMI and insulin secretion levels, vary among the various ethnic groups with different genetic backgrounds or life styles [5]. Furthermore, most of the susceptibility loci identified through GWAS to date are likely to affect insulin secretion and β-cell function, while a few are potentially involved in insulin action [30,31].

In the present study, we analyzed the relationships between the genotypes of 17 susceptibility loci identified by GWAS and T2DM in a Japanese population-based case-control study. We also examined the combined effect of the cumulative number of risk alleles of the T2DM and obesity/overweight for the development of T2DM.

## Methods

### Subjects

This is a population based case-control study. The participants for this study were recruited from Japanese who underwent a routine medical check-up at a medical center near the University of Shizuoka on 2005. We have selected men (N = 6094) under 65 years of age as subjects in this study. Of these, 378 men (6.2%) were diagnosed with T2DM by physicians according to the World Health Organization (WHO) diagnostic criteria for T2DM (http://www.who.int/diabetes/publications/diagnosis_diabetes2006/en/). Of them, 333 men were included as T2DM subjects in this study, as complete genotype information on all 17 SNPs analyzed in this study was obtained. For the control subjects, 417 men (aged 45 to 65 years) were randomly selected from all subjects according to the following criteria: (1) their fasting plasma glucose levels were under 100 mg/dl (5.6 mmol/1), (2) their HbA1c levels were under 5.8%, and (3) complete genotype information on all 17 SNPs was obtained. All subjects provided written informed consent to participate in this study, and the study was approved by the Ethics Committee of the University of Shizuoka.

After overnight fasting, blood was collected from each subject. The clinical characteristics of the subjects were determined according to the medical check-up protocol.

### DNA analysis

Genomic DNA was isolated from peripheral leucocytes by the phenol extraction method. We analyzed the genotypes of 17 SNPs in or near 17 susceptibility loci for T2DM. At first, we selected 10 susceptibility loci (*SLC30A8, CDKN2A/B, KCNJ11, IGF2BP2, CDKAL1, HNF1B, HHEX, FTO, TCF7L2, PPARG*), which had been detected in the first stage of GWAS for Caucasian [14], and four loci *(CDC123, ADAMTS9, TSPAN8, JAFZ*), which had been detected in a meta-analysis of three large GWAS [15], because they had been confirmed in several replication studies [17-23]. In addition, three SNPs (*KCNQ1, C2CD4A, UBE2E2*), which were discovered in GWAS using Japanese people, were examined [24,25,28]. However, three loci (*WFS1, NOTCH2, THADA*) which were also ascertained to be associated with T2DM in the first stage GWAS or the meta-analysis of three large GWAS [14,15], were excluded from this analysis due to low minor allele frequencies in Japanese (< 0.03) [17,23]. Although more and more susceptibility loci for T2DM are being identified, we have not yet examined such new susceptibility loci.

The genotypes of these 17 susceptibility loci were determined for each subject using the PCR-restriction fragment length polymorphism method. The genotype call rate for each SNP was > 95%. The genotype distributions of these 17 SNPs were in Hardy-Weinberg equilibrium (*P *> 0.05).

### Statistical analyses

Each risk allele was defined as the allele associated with increased risk of T2DM in previous studies [23,28]. The each allele-specific odds ratios (ORs) with 95% confidence intervals (CIs) and *P*-values for T2DM were calculated under the assumption of an additive model using logistic regression analysis, adjusting for age and BMI. In addition, the cumulative number of risk alleles was counted, in which individuals homozygous for non-risk alleles were coded as 0, heterozygous individuals were coded as 1 and individuals homozygous for the risk alleles were coded as 2, with the assumption that each risk allele acted independently and contributed equally to the risk of T2DM. The effects of the cumulative number of risk alleles, BMI or obesity/overweight, and the interaction between the cumulative number of risk alleles and BMI or obesity/overweight on the prevalence of T2DM were assessed using multivariate logistic regression analysis.

Statistical analyses were performed using the JMP software package (SAS Institute, Cary, NC). The power to detect an association between each SNP and T2DM was estimated under current sample size and minor allele frequency observed in this study using "Quanto" (http://hydra.usc.edu/gxe/), assuming OR = 1.2 and α level = 0.05 (one-sided). The Cochran-Armitage test was used to examine the trend of an increase in the OR by an increasing number of the risk alleles.

## Results

The characteristics of our subjects are presented in Table 1. Of the total 6094 subjects, the prevalence of diabetes, obesity, and being overweight were 6.2%, 2.6%, and 27.0%, respectively. The BMI, blood pressure, serum triglyceride, glucose and HbA1c were higher, and HDL-cholesterol was lower, in subjects with T2DM compared with control subjects.

**Table 1:** Characteristics of the study subjects

	All subjects	T2DM	Control	*P-*value
	n = 6094	n = 333	n = 417	
Age (years)	49.9 ± 8.3	54.4 ± 6.4	53.7 ± 5.1	0.19
BMI (kg/m2)	23.7 ± 3.0	25.0 ± 3.6	23.1 ± 2.7	< 0.0001
SBP (mmHg)	119.9 ± 15.1	126.3 ± 15.6	120.2 ± 16.2	< 0.0001
DBP (mmHg)	76.6 ± 11.4	79.7 ± 11.0	76.6 ± 11.8	0.0004
Total-cholesterol (mg/dl)	211.9 ± 33.9	212.7 ± 35.0	211.1 ± 31.9	0.77
LDL-cholesterol (mg/dl)	130.0 ± 30.2	131.2 ± 29.8	130.3 ± 29.9	0.65
HDL-cholesterol (mg/dl)	57.8 ± 16.3	54.0 ± 16.1	58.7 ± 16.4	0.0002
Triglyceride (mg/dl)	140.0 ± 107.0	160.5 ± 146.7	134.9 ± 110.6	0.012*
Glucose (mg/dl)	100.5 ± 19.8	153.8 ± 40.7	91.8 ± 4.9	< 0.0001*
HbA1c (%)	5.3 ± 0.76	7.3 ± 1.5	5.1 ± 0.33	< 0.0001
T2DM (%)	6.2	100	0	-
Obesity (BMI ≧ 30) (%)	2.6	6.9	1.0	< 0.0001
Overweight (BMI ≧ 25) (%)	27.0	37.8	21.6	< 0.0001
Hypertension (%)	13.5	29.3	13.2	0.0004
Current smorker (%)	39.8	44.6	41.4	0.62

*Statistical test for triglyceride and glucose levels were calculated on log-transformed values

*P*-values between T2DM and contol groups were calculated by *t*-test or *χ*^2^-test

Data are expressed as mean ± SD or percentage

We then analyzed the relationships between common genetic variants of 17 T2DM susceptibility loci that have been previously detected by GWAS and T2DM in Japanese men. Table 2 shows the risk allele frequencies of each SNP, and risk allele-specific OR, *P*-value and estimated power to detect the association, assuming OR = 1.2. The ORs and P-values were adjusted for age and BMI in a logistic regression analysis. Four risk alleles for *SLC30A8, CDKN2A/B, CDC123*, and *KCNQ1 *were significantly associated with T2DM (*P *< 0.05), although three of them were not significant when Bonferroni's correction for multiple testing applied (significance level, 0.05/17 = 0.0029). Of the remaining loci, except for *PPARG, ADAMTS9, TSPAN8 *and *JAZF1*, the risk allele frequencies of T2DM subjects were higher than that of control subjects, although not statistically significant.

**Table 2:** Individual effects of 17 risk alleles of the susceptibility loci on T2DM

			Risk allele frequency					
							
Locus	db SNP	Risk allele	T2DM	Control	OR	(95% CI)	*P*-value	Power
*SLC30A8*	rs13266634	C	0.64	0.57	1.95	(1.26-3.04)	**0.0026**	0.41
*CDKN2A/B*	rs10811661	A	0.61	0.54	1.96	(1.26-3.08)	**0.0030**	0.41
*CDC123*	rs11257622	C	0.26	0.21	1.99	(1.21-3.29)	**0.0068**	0.31
*KCNQ1*	rs2237892	C	0.64	0.58	1.85	(1.18-2.93)	**0.0073**	0.40
*KCNJ11*	rs5219	T	0.41	0.37	1.48	(0.96-2.28)	0.074	0.40
*C2CD4A*	rs7172432	A	0.59	0.54	1.48	(0.95-2.31)	0.081	0.41
*IGF2BP2*	rs4402960	T	0.34	0.30	1.45	(0.91-2.31)	0.12	0.37
*CDKAL1*	rs10946403	G	0.50	0.46	1.35	(0.88-2.08)	0.17	0.42
*HNF1B*	rs7501939	T	0.35	0.32	1.35	(0.85-2.14)	0.20	0.38
*HHEX*	rs1111875	G	0.30	0.28	1.25	(0.78-1.98)	0.35	0.36
*UBE2E2*	rs7612463	C	0.84	0.83	1.31	(0.73-2.36)	0.37	0.25
*FTO*	rs8050136	A	0.22	0.20	1.26	(0.73-2.15)	0.40	0.30
*TCF7L2*	rs7903146	T	0.06	0.05	1.39	(0.56-3.42)	0.48	0.12
*PPARG*	rs1801282	C	0.97	0.97	1.49	(0.44-5.34)	0.53	0.09
*ADAMTS9*	rs4607103	C	0.60	0.60	0.81	(0.52-1.25)	0.35	0.40
*TSPAN8*	rs7961581	C	0.19	0.19	0.95	(0.54-1.69)	0.87	0.29
*JAZF1*	rs864745	T	0.79	0.80	0.93	(0.55-1.57)	0.78	0.28

Odd ratios and *P*-values were adjusted for age and BMI under the additive model

Power to detect association was estimated under current sample size and minor allele frequency, assuming OR = 1.2

**Figure 1:**
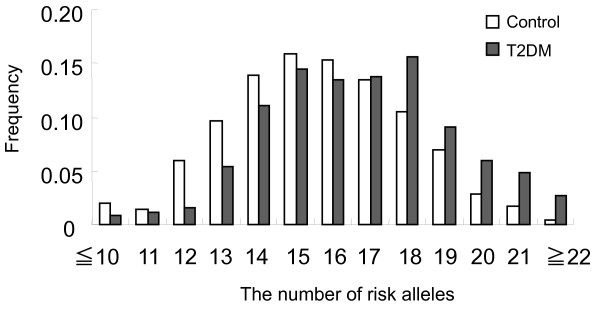
**Distribution of the cumulative number of risk alleles of 17 T2DM susceptibility loci in T2DM and control subjects**.

Next, we calculated the cumulative number of these 17 risk alleles that each subject possessed. The distribution of the cumulative number of risk alleles in T2DM subjects shifted to the right compared with that of the control subjects. The mean risk allele number in T2DM subjects (16.7 ± 2.5) was significantly higher than that in control subjects (15.6 ± 2.4) (*P *< 0.0001, *t*-test) (Figure 1). Multivariable regression analyses indicated that both the cumulative number of risk alleles and BMI or obesity/overweight were important predictors of T2DM (*P *< 0.0001). In addition, we found interactions between the cumulative number of risk alleles and BMI or obesity/overweight for developing T2DM (*P *= 0.0080 or *P *= 0.015, respectively) (Table 3). And the cumulative number of risk alleles was not an independent predictor of T2DM when the interaction between the number of risk alleles and obesity/overweight was incorporated in the regression model as a covariate (*P *= 0.81) (Table 3, Model 4).

**Table 3:** Multivariable regression analyses for T2DM

	Model 1			Model 2		
R^2^	0.10			0.11		
intercept	-10.43			-10.52		

Determinant variables	β-coefficient	SE	P	β-coefficient	SE	P

No. of risk alleles	0.18	0.033	< 0.0001	0.18	0.033	< 0.0001
BMI	0.21	0.028	< 0.0001	0.22	0.029	< 0.0001
Age	0.04	0.014	0.0038	0.04	0.014	0.0036
BMI + No. of risk allele				-0.029	0.011	0.0080

	Model 3			Model 4		

R^2^	0.089			0.097		
intercept	-4.41			-1.11		

Determinant variables	β-coefficient	SE	P	β-coefficient	SE	P

No. of risk alleles	0.18	0.032	< 0.0001	-0.026	0.11	0.81
Obesity/overweight	-1.22	0.20	< 0.0001	-1.19	0.22	< 0.0001
Age	0.04	0.014	0.0047	0.04	0.014	0.0047
Obesity/overweight + No. of risk allele				0.26	0.11	0.015

Model 1 included the number of risk alleles, BMI and age as covariates

Model 2 included the number of risk alleles, BMI, age and the interaction between the number of risk alleles and BMI as covariates

Model 3 included the number of risk alleles, obesity/overweight and age as covariates

Model 4 included the number of risk alleles, obesity/overweight, age and the interaction between the number of risk alleles and obesity/overweight as covariates

*SE *standard error

To examine the relationships between an increased number of risk alleles and T2DM, we stratified the subjects into four groups by quartiles of the risk allele numbers (Q1, ≤ 14; Q2, 15, 16; Q3, 17, 18; and Q4, ≥ 19, respectively). The OR associated with each quartile group compared with the reference group (Q1) are shown in Table 4. An increased number of risk alleles was associated with an increased risk of T2DM (*P *< 0.0001 for trend).

**Table 4:** Relationships between increased number of risk alleles and T2DM

Quartiles of risk alleles	All subjects (n = 750)*					Obese/overweight (n = 243)**					Non-obese (n = 507)**				
	
	n					n					n				
												
	T2DM	Control	OR	(95% CI)	*P*-value	T2DM	Control	OR	(95% CI)	*P*-value	T2DM	Control	OR	(95% CI)	*P*-value
Q1	67	137	1	reference		36	25	1	reference		31	112	1	reference	
(≦ 14)															
Q2	93	130	1.42	(0.93-2.19)	0.11	43	26	1.15	(0.57-2.35)	0.69	50	104	1.71	(1.01-2.91)	0.045
(15, 16)															
Q3	98	100	1.95	(1.27-2.99)	0.002	40	31	0.89	(0.44-1.79)	0.75	58	69	3.13	(1.85-5.41)	< 0.0001
(17, 18)															
Q4	75	50	3.14	(1.93-5.14)	< 0.0001	30	12	1.77	(0.77-4.23)	0.18	45	38	4.20	(2.33-7.66)	< 0.0001
(≧ 19)															

				P for trend	< 0.0001				P for trend	0.88				P for trend	< 0.0001

*ORs and *P*-values on T2DM for all subjects were adjusted with age and BMI

**ORs and *P*-values on T2DM for obese/overweight and non-obese subjects were adjusted with age

Furthermore, to test for an interaction between the number of risk alleles and obesity/overweight on T2DM risk, we divided the subjects into two groups; the obese/overweight group (BMI ≥ 25 kg/m^2^) and the non-obese group (BMI < 25 kg/m^2^). Significant association between an increased number of risk alleles and an increased risk of T2DM was observed only in the non-obese group (*P *< 0.0001 for trend), and not in the obese/overweight group (*P *= 0.88 for trend) (Table 4). In addition, we analyzed the association between obesity/overweight and T2DM according to quartiles of the risk allele numbers. Obesity/overweight was a strong predictor of T2DM in our Japanese subjects (*P *< 0.0001), however, for the subjects in Q3 and Q4 (≥ 17 risk allele), obesity/overweight was not a significant risk factor for T2DM (*P *> 0.05) (Table 5). These findings indicate that there is an etiological heterogeneity of T2DM between obese/overweight and non-obese subjects.

**Table 5:** Effect of obesity/overweight on T2DM according to quartiles of risk allele numbers

Quartiles		T2DM	Contrrol	OR	(95% CI)	*P-*value
of risk alleles		(n = 333)	(n = 417)			
All subjects	obese	149	94	2.78	(2.10-3.72)	< 0.0001
	non-obese	184	323			
Q1	obese	36	25	5.31	(2.79-10.33)	< 0.0001
(≦ 14)	non-obese	31	112			
Q2	obese	43	26	3.59	(1.98-6.61)	< 0.0001
(15, 16)	non-obese	50	104			
Q3	obese	40	31	1.61	(0.89-2.93)	0.11
(17, 18)	non-obese	58	69			
Q4	obese	30	12	2.18	(0.99-5.02)	0.052
(≧ 19)	non-obese	45	38			

ORs and *P*-values were adjusted with age

## Discussion

In this population-based case-control study, we have shown that the cumulative number of risk alleles based on 17 susceptibility loci for T2DM, identified through GWAS in Caucasian and Asian populations, was a significant risk factor in a Japanese population, although the effect of each risk allele was relatively small. Obesity is a very important risk factor for T2DM, however, many obese people do not develop T2DM, while many non-obese people do. In our population-based study, 9.5% of subjects in the obese/overweight group, and 4.7% of subjects in the non-obese group, had T2DM (data not shown). Impaired insulin resistance and insulin secretion are key determinants of T2DM development. It is well known that insulin resistance is associated with obesity, but insulin secretion is not affected by body constitution [2,7].

The possibility of etiological heterogeneity of T2DM between obese/overweight and non-obese subjects cannot be overlooked. In fact, a significant association between an increased number of risk alleles and an increased risk of T2DM was observed only in non-obese group (*P *< 0.0001 for trend), and an increased number of risk alleles was not a significant risk factor for subjects in the obese/overweight group in this study (*P *= 0.88 for trend). However, the power of our study is insufficient to detect positive association in obese/overweight group due to the small sample size. It is necessary to confirm these finding in another large population.

It was reported previously that risk alleles affecting insulin action more significantly increase T2DM susceptibility in obese individuals, while risk alleles affecting insulin secretion confer a T2DM risk in non-obese individuals [32]. Most of the susceptibility loci analyzed in the present study appear to influence β-cell function, such as insulin secretion or β-cell proliferation, which reasonably explains why the association between an increased number of risk alleles and an increased risk of T2DM was observed only in our non-obese subjects. Furthermore, it is possible that the ability of insulin secretion is weak for subjects with an increased number of risk alleles. Unfortunately, we were unable to examine serum insulin levels of our subjects, and thus, the relationship between an increased number of risk alleles and insulin secretion in our subjects remains unclear. Further examination is required to determine β-cell function in our subjects.

Recently, several studies indicates the cumulative number of risk alleles is an important risk factor for T2DM in Asian population [20,22,33], moreover there is increasing interest that knowledge about genetic risk factors may be used to predict the risk of complex disorders such as T2DM [20,22,33-35]. Our data indicate that both the cumulative number of risk alleles and obesity/overweigh are important risk factors for T2DM, but that obesity/overweight is not a significant risk factor of T2DM in subjects with many risk alleles (Q3 and Q4; risk allele number ≥ 17). Most T2DM patients in Japan are characterized by a low BMI, it might be useful for Japanese population to count the number of risk alleles of susceptible loci to improve identification of high-risk subjects. However, our study is a population-based case-control study; therefore, we are unable to determine the predictive power of such susceptibility loci. Further prospective studies are required to translate such knowledge about genetic risk factors into clinical practice for prediction and prevention of T2DM in the general population.

## Conclusions

We have shown that the cumulative number of risk alleles based on 17 susceptibility loci for T2DM, identified through GWAS in Caucasian and Asian populations, was a significant risk factor in a Japanese population, although the effect of each risk allele was relatively small. In addition, the association between an increased number of risk alleles and an increased risk of T2DM was observed only in non-obese group, and not in obese/overweight group. These data indicate that there is an etiological heterogeneity of T2DM between obese/overweight and non-obese subjects. In future, knowledge about genetic risk factors might be used in clinical practice for prediction and prevention of T2DM in the general population.

## Abbreviations

*SLC30A8*: Solute carrier family 30 (zinc transporter) member 8; *CDKN2A/B*: Cyclin-dependent kinase inhibitor 2A and B (melanoma p16, inhibits CDK4); *CDC123*: Cell division cycle 123 homolog (S. cerevisiae); *KCNJ11*: Potassium inwardly-rectifying channel subfamily J, member 11; *IGF2BP2*: Insulin-like growth factor 2 mRNA binding protein 2; *CDKAL1*: CDK5 regulatory subunit associated protein 1-like 1; *HNF1B*: HNF1 homeobox B; *HHEX*: Hematopoietically expressed homeobox; *FTO*: Fat mass and obesity associated; *TCF7L2*: Transcription factor 7-like 2 (T-cell specific HMG-box); *PPARG*: Peroxisome proliferator-activated receptor γ; *ADAMTS9*: ADAM metallopeptidase with thrombospondin type 1 motif 9; *TSPAN8*: Tetraspanin-8; *JAFZ1*: JAZF zinc finger 1; *KCNQ1*: Potassium voltage-gated channel KQT-like subfamily, member 1; *UBE2E2*: Ubiquitin-conjugating enzyme E2E 2; *C2CD4A*: C2 calcium-dependent domain containing 4A; PCR: Polymerase chain reaction; RFLP: Restriction fragment length polymorphism; SNP: Single nucleotide polymorphism; BMI: Body mass index; SBP: Systolic blood pressure; DBP: Diastolic blood pressure; LDL: Low-density lipoprotein; HDL: High-density lipoprotein; HbA1c: Hemoglobin A1c.

## Competing interests

The authors declare that they have no competing interests.

## Authors' contributions

KYK managed the study, and carried out the genetic analyses, the genotyping experiments, drafting the manuscript. NM, SA, SN, TI carried out the genotyping experiments. NK and TG participated in the design of the study and recruitment of study subjects. All authors read and approved the final manuscript.

## Pre-publication history

The pre-publication history for this paper can be accessed here:

http://www.biomedcentral.com/1471-2350/13/11/prepub
